# The Role of Distance and Quality on Facility Selection for Maternal and Child Health Services in Urban Kenya

**DOI:** 10.1007/s11524-017-0212-8

**Published:** 2017-12-21

**Authors:** Veronica Escamilla, Lisa Calhoun, Jennifer Winston, Ilene S. Speizer

**Affiliations:** 1 Carolina Population Center, University of North Carolina (UNC), Chapel Hill, NC USA; 2 Department of Obstetrics and Gynecology - Division of Global Women’s Health, UNC, Chapel Hill, NC USA; 3 Gillings School of Public Health, Department of Maternal and Child Health, UNC, Chapel Hill, NC USA

**Keywords:** Urban reproductive health, Health service access, Kenya

## Abstract

Universal access to health care requires service availability and accessibility for those most in need of maternal and child health services. Women often bypass facilities closest to home due to poor quality. Few studies have directly linked individuals to facilities where they sought maternal and child health services and examined the role of distance and quality on this facility choice. Using endline data from a longitudinal survey from a sample of women in five cities in Kenya, we examine the role of distance and quality on facility selection for women using delivery, facility-based contraceptives, and child health services. A survey of public and private facilities offering reproductive health services was also conducted. Distances were measured between household cluster location and both the nearest facility and facility where women sought care. A quality index score representing facility infrastructure, staff, and supply characteristics was assigned to each facility. We use descriptive statistics to compare distance and quality between the nearest available facility and visited facility among women who bypassed the nearest facility. Facility distance and quality comparisons were also stratified by poverty status. Logistic regression models were used to measure associations between the quality and distance to the nearest facility and bypassing for each outcome. The majority of women bypassed the nearest facility regardless of service sought. Women bypassing for delivery traveled the furthest and had the fewest facility options near their residential cluster. Poor women bypassing for delivery traveled 4.5 km further than non-poor women. Among women who bypassed, two thirds seeking delivery and approximately 46% seeking facility-based contraception or child health services bypassed to a public hospital. Both poor and non-poor women bypassed to higher quality facilities. Our findings suggest that women in five cities in Kenya prefer public hospitals and are willing to travel further to obtain services at public hospitals, possibly related to free service availability. Over time, it will be important to examine service quality and availability in public sector facilities with reduced or eliminated user fees, and whether it lends itself to a continuum of care where women can visit one facility for multiple services reducing travel burden.

**Electronic supplementary material:**

The online version of this article (10.1007/s11524-017-0212-8) contains supplementary material, which is available to authorized users.

## Background

The 2030 Sustainable Development Goals call for universal health care, which will require that services are available and accessible for those most in need of maternal and child health services [1]. In Kenya, policies to increase health facility care include removal of patient fees at dispensaries and health centers, and provision of free family planning and maternal health services, including delivery, in all public facilities [2]. As policy makers work to remove financial barriers to access, it is important to understand what other barriers exist, such as distance to facilities and perceived quality of care. These barriers may influence where women seek maternal and child health services, and ultimately the goal of universal health care access.

In rural settings, increased distance can reduce facility use for reproductive health services including family planning and delivery [3–7], or child health services [8, 9]. In rural Ghana, contraceptive use was significantly lower among women living 2 km or more from a facility compared to women living within 2 km of a facility [3]. Another study in rural Ghana found that facility delivery was less likely among women living further than 1 km from a facility compared to women living within 1 km of a facility [10]. Besides distance, women often bypass the facility closest to their home due to lack of services or poorly perceived service quality [11–14]. Research in rural Kenya found that service availability at the nearest facility was more influential than distance when seeking delivery or child health services [15]. Bypassing nearby facilities when seeking maternal health services is also influenced by household wealth and cost of services [13, 15].

The relationship between distance and facility selection in urban settings is less clear as women have more health service options within reasonable travel distance. Factors such as market or employment location may also influence health facility selection. In a dense urban setting in Senegal, women were willing to travel further to obtain family planning services from higher quality facilities [16]. In urban Sierra Leone, few women cited facility distance compared to reputation or cost as a primary consideration when selecting health providers for their children [17]. The study took place in adjacent neighborhoods with multiple clinics and hospitals within a 1 km radius, potentially attenuating the role of distance [17]. The relationship between distance and facility selection for delivery in urban settings is also complex. Health service availability and access does not ensure use. While urban women are more likely than rural women to deliver in facilities [18], the percentage of urban women delivering outside of a facility remains high. A Nigeria DHS study found that nearly half of all urban women gave birth outside of a facility [19]. In an urban settlement in Lagos, Nigeria, women had multiple facility options within the city, but over half (51.4%) delivered outside of facilities [20].

Poverty further complicates the relationship between health service access and bypassing behaviors in urban environments. Urban poor often live on city outskirts and must travel further for health services [21]. Inability to pay service fees or the cost of consumables poses a challenge for many women who would prefer to seek another provider if cost was not an issue [17]. Further, insufficient funds to cover fees often result in women choosing to deliver at home [20] or treat a sick child at home rather than visit a health facility [22]. Urban poor living in informal settlements face added challenges of insufficient quality health care options [23]. Studies from two informal settlements in Nairobi, Kenya, found that women did not consider distance or transport a hindrance to visiting the nearest facility within the settlement [24]. However, facilities within informal settlements are often private making cost a challenge for obtaining care [25, 26] and are often poorly equipped to provide adequate services [27, 28]. Focus groups from an informal settlement in Nairobi, Kenya, found that poor women recognize the safety of hospital delivery for complications but prefer home births due to challenges with traveling outside the settlement to reach the main road for transport, transport costs and facility-related costs, and perceived negative provider treatment [29].

The objective of this study was to examine the role of distance and service quality on facility bypassing and selection in complex urban settings. We selected multiple services, including delivery, facility-based methods (e.g., implant, IUD, sterilization, and injectable), and child health visits to examine heterogeneity of factors influencing facility selection. Few studies have been able to directly link individuals seeking maternal and child health services to the facility where they sought care and examine distance and quality of facilities visited and facilities not visited [30]. Our study compares facility distance and quality between the nearest available and selected facilities, specifically among women who chose to bypass the nearest available facility. We then stratify the comparison by poor and non-poor women who bypassed the nearest facility. By focusing on a complex urban setting where women have multiple facility options of various types (e.g., public/private, hospitals, clinics) within walking distance, we can better understand facility characteristics that may encourage women to use the same facility for multiple services as care continuity benefits maternal and child health [31]. A better understanding of how distance and quality interact is important for improving facility use and reducing patient travel burden.

## Data and Methods

### Study Population

We utilized data from the Measurement, Learning & Evaluation (MLE) Project in Kenya. As part of the evaluation of the Kenya Urban Reproductive Health Initiative (called Tupange), longitudinal data were collected from women at baseline (2010) and endline (2014) in five urban centers of Kenya: Kakamega, Kisumu, Machakos, Mombasa, and Nairobi.

At baseline (2010), a representative sample of women from each city was obtained using a multistage sampling design. Government census enumeration areas served as primary sampling units (PSU), and 30 households were randomly sampled from each selected PSU. In three cities (Nairobi, Mombasa, and Kisumu), the sampling frame indicated if a PSU was predominately formal settlements (non-slum) or informal settlements (slum). Oversampling was conducted to include a larger sample of slum PSU in these cities; weights are used to make the sample representative of each city. After a household interview, all eligible women (aged 15–49 years) were invited to participate in the survey after providing informed consent. Women’s survey data include demographic information, modern contraceptive use, and facility selection for multiple maternal and child health services. In addition, global positioning system (GPS) coordinates were collected for each PSU centroid location. A total of 8932 women were interviewed at baseline across the five cities.

At endline (2014), women who still resided in their baseline study city and those who moved within one of the five study cities were eligible for endline interview; GPS coordinates were collected for the new residential location if a woman moved. In total, we found 59% of women at endline (*n* = 5217). This study focuses on women found at endline who visited a health facility for delivery, facility-based contraception, and child health services in the period of observation related to each service. Respondents were asked the name and approximate location of the facility where they sought each service. Responses were coded using the MLE facility study list (see below) and the Kenya Master Facility List.

Of the 5217 women surveyed at endline, 1350 (25.9%) gave birth in the last 2 years. Among those who gave birth, 1168 delivered in a health facility, of which 68.5% (*n* = 800) delivered in a facility surveyed by MLE.

A total of 2814 women reported modern contraceptive use at endline, of which 75.9% (*n* = 2135) reported using a facility-based method. Facility-based methods require visiting a health facility and thus are the focus of the contraceptive use analysis. Of those using a facility-based method, 68.7% (*n* = 1467) obtained their method from a facility surveyed by MLE.

A total of 3670 women had one or more children living at home at endline. Of those women, 1595 took their child to a facility for a health visit in the past 3 months, of which 69.7% (*n* = 1112) specifically visited a facility in the MLE facility study.

### MLE Facility Study Data

At baseline (2010) and endline (2014), the MLE project also surveyed public and private health facilities as part of the Tupange evaluation. A complete census of facilities offering reproductive health services was conducted in Kakamega, Kisumu, and Machakos. In Mombasa and Nairobi, it was not possible to include a census of facilities given the number of facilities in these cities. Therefore, in these cities, all public sector facilities were included as well as all private facilities where the intervention program (Tupange) planned to implement activities. In addition, we included other private facilities based on those facilities women reported visiting for family planning and maternal and child health services during the baseline survey. At endline, 377 public and private facilities were surveyed. Facility surveys captured information on services provided, trained staff availability, and contraceptive method availability and method stockouts, among other factors. For this study, we focus on facilities surveyed at endline that offered at least one of the following: delivery, facility-based contraceptives, or child health services. We included the appropriate facilities that offered the relevant service for each analysis.

Euclidean (straight-line) distances were measured between the household cluster location and the MLE surveyed facility where women reported seeking care for delivery, facility-based contraception, or child health visits. Distances were also measured between household clusters and the nearest MLE surveyed facility offering the specific service sought to compare quality between the visited and nearest bypassed facility. For women who moved at endline, distance was measured from their household location to the facility they visited and the nearest available facility.

We calculated the total number of MLE surveyed facilities offering delivery, facility-based contraceptive methods, and child health services within 3 km of a woman’s residential cluster location or endline residence location (for those who moved). While previous studies have identified living within 2 km of a facility as a significant factor for choosing to deliver in a facility [7, 32], we use a 3 km threshold to better understand facility options within a range of walking distances (0.5 to 3 km). All distance measures were calculated using ArcGIS 10.2.2.

### Statistical Analysis

A categorical wealth index variable representing a composite score of ownership of household assets and household structure characteristics was generated using principle components [33]. The wealth index was divided into quintiles with 5 reflecting richest and 1 reflecting poorest. The wealth index was converted to a binary variable to examine differences between poor (scores 1–2) and non-poor (scores 3–5) women.

A facility quality index variable was created using principle component analysis. The index comprised multiple factors representing facility accessibility, staff availability, and infrastructure and hygienic resources. Factors included previously identified indicators of facility quality such as hours of operation and staffing [6], and availability of piped water and a private exam room [34]. We estimated the ratio of staff (i.e., physicians, nurses, community health workers) per 1000 family planning and maternal and child health patients to gain a better estimate of facility capacity.^1^ We also included hygiene related supplies such as liquid soap and latex gloves because while patients may not notice these supplies, they reflect hygienic practices of the clinic that represent quality (full list of factors included in PCA shown in Supplement A). The quality index was divided into quintiles with 5 reflecting highest quality and 1 reflecting lowest quality.

Descriptive statistics (proportions and means) were used to better understand differences between the quality of the nearest available facility and the visited facility among women who bypassed. Comparisons were also made between poor and non-poor women who bypassed the nearest facility. A sensitivity analysis examining bypassing and facility quality was restricted to women living in cities with a facility census (Kisumu, Kakamega, Machakos) to determine if results differed with the full set of facility options. Analyses were conducted using the “svy” function in Stata v.14.2 to adjust for weights. Statistically significant differences were assessed using the Adjusted Wald test and the Rao-Scott correction F statistic for Pearson chi-square.

Logistic regression models were then built to better understand why women bypass the nearest facility. Separate models were undertaken for the three outcomes, delivery, facility-based contraceptive use, and child health services. Predictors of interest were the quality index of the nearest facility and distance to the nearest facility. Covariates included level of the nearest facility (hospital, health center, dispensary) and whether the nearest facility was public or private. Household ownership of a vehicle was also tested as a covariate for each outcome and remained in the model if it was significant at *p* < 0.1 in unadjusted models. We tested a number of additional covariates for the facility-based contraceptive model, including stockout of methods in the past year, total method types offered, and free methods offered if the nearest facility was private. Only covariates significant at *p* < 0.1 in unadjusted models remained in the facility-based contraceptive model. All models adjusted for age, wealth, education, marital status, and city of residence. All models were weighted to account for differential selection probabilities and differential non-response.

Cost data were not available for all services; however, the public variable served as a proxy for cost because all public facilities offer family planning and maternal and child health services for free. Cost information for private facilities was not available for delivery or child health services.

## Results

Among women surveyed at endline who reported visiting an MLE facility for a service, 800 delivered a baby, 1467 obtained facility-based contraception, and 1112 reported child health visits (Table 1). More than half of women visiting an MLE facility for any service were between 25 and 34 years old. Wealth and education distributions were similar across all services received (Table 1). Among women receiving facility-based contraception at an MLE facility, the majority received an injection (47.8%) followed by an implant (34.3%), with low levels for sterilization (9.3%) and IUD (8.7%).

**Table 1:** Percentage distribution of sociodemographic characteristics of women surveyed at endline by service received

	Delivery reported in last 2 years	Facility-based contraceptive use at endline	Reported child health visit in past 3 months
Total women visit MLE surveyed facility for service*	*N* = 800	*N* = 1467	*N* = 1112
Age group			
15–19	0.9	1.5	0.5
20–24	22.0	13.0	15.4
25–29	36.5	30.1	35.0
30–34	25.4	24.6	24.3
35–39	11.6	15.1	13.7
40–49	3.6	13.3	9.7
Married/live with partner	82.5	84.9	83.5
Education			
None	1.9	2.0	2.4
Primary	40.0	51.7	44.3
Secondary	35.3	31.2	33.8
≥ Some college	22.6	15.1	19.3
Wealth index^§^			
Poorest	25.3	32.2	29.3
Poor	21.9	21.8	20.1
Middle	19.8	20.3	23.0
Rich	18.7	15.6	15.7
Richest	14.3	10.1	11.9
City of residence			
Nairobi	21.0	16.7	14.8
Mombasa	14.6	10.5	12.6
Kisumu	19.8	21.5	24.5
Machakos	24.3	31.0	26.5
Kakamega	20.4	20.4	21.6
Informal settlement residence	21.0	20.1	22.0

Percentages are weighted

*Denominator for all variables

^§^Wealth index generated using principal component analysis represents ownership of household assets

Of the 377 facilities surveyed at endline, 45.2% offered delivery services, 95.9% offered facility-based contraceptives, and 96.4% offered child health services. Characteristics of the facilities offering these services are presented in Supplement A. Facilities included public hospitals, health centers, and dispensaries, as well as private health centers and faith-based and for-profit hospitals.

The majority of women reported bypassing the nearest facility for each service (Table 2). Women traveled furthest for delivery and had the fewest facility options for delivery within 0.5 to 3 km surrounding their home location. Women visiting facilities for facility-based contraception and child health visits traveled similar. Women seeking child health services had the most facility options within 3 km of their home cluster. Sensitivity analyses restricted to cities with a complete facility census (Kakamega, Kisumu, Machakos) yielded similar results (Supplement B).

**Table 2:** Bypassing behavior and facility availability among women surveyed at endline who visited a MLE surveyed facility for delivery, facility-based contraception, or child health visits

	Delivery reported in last 2 years *N* = 800	Facility-based contraceptive use at endline *N* = 1467	Reported child health visit in past 3 months *N* = 1112
% visit nearest MLE facility offering service	12.0	16.1	15.8
Mean distance traveled among women visiting nearest MLE facility	1.2 km	0.8 km	0.8 km
% bypass nearest MLE facility offering service	88.0	83.9	84.3
Mean distance traveled among women bypassing nearest MLE facility	10.0 km	6.2 km	5.8 km
Mean total MLE facilities offering service within 0.5 km (range)	0.4 (0–4)	0.8 (0–11)	0.9 (0–11)
Mean total MLE facilities offering service within 1 km (range)	1.4 (0–8)	2.8 (0–14)	3.1 (0–15)
Mean total MLE facilities offering service within 2 km (range)	4.2 (0–17)	7.8 (0–23)	8.6 (0–26)
Mean total MLE facilities offering service within 3 km (range)	7.2 (0–18)	14.2 (0–25)	15.6 (0–32)

Mean values and percentages are weighted

On average, women who bypassed traveled significantly further than the nearest available facility regardless of service sought (Table 3). Women who bypassed for delivery or facility-based contraceptives tended to visit facilities that were higher quality then the nearest facility (Table 3). The majority of women who bypassed for delivery visited a public hospital, and nearly half of the women seeking facility-based contraceptives or child health services bypassed to a public hospital. For the majority of women, the closest facility was a private facility; however, fewer than 25% bypassed this facility to visit another private facility. Private facility visits were lowest among women seeking child health services (16.5%). More facilities visited by women for facility-based contraceptives reported stockouts in the last year compared to the nearest bypassed facilities (Table 3). This may relate to greater use and demand in these facilities. Results were similar for sensitivity analyses restricted to cities with a complete facility census (Supplement B).

**Table 3:** Quality comparison of MLE facility visited and MLE facility bypassed by service received among women who bypassed the nearest available facility offering the service

Facility characteristics	Facility visited	Nearest facility
Delivery reported in last 2 years (*N* = 702**)**		
Mean distance to facility offering delivery (km)	10.0	1.6^§^
Facility quality index		
Lowest	0.1	5.0^§^
Low	0.2	16.8
Middle	6.2	6.4
High	10.8	30.7
Highest	82.7	41.1
Percent public facility	76.0	36.7
Percent public hospital	69.4	6.0^§^
Percent private facility	24.0	63.4
Facility-based contraceptive use at endline (*N* = 1227)		
Mean distance to facility offering facility-based methods (km)	6.2	1.1^§^
Facility quality index, %		
Lowest	3.5	27.1^§^
Low	7.6	20.5
Middle	17.1	12.5
High	19.9	21.9
Highest	51.9	18.0
Percent public facility	78.2	20.9**
Percent public hospital	46.8	1.7^§^
Percent private facility	21.8	79.1**
Percent private facility offering some facility-based methods free	3.2	8.6**
Mean total facility-based methods offered	3.4	2.6^§^
Percent report facility-based method stockout in past year	30.1	21.3*
Reported child health visit in past 3 months (*N* = 940)		
Mean distance to facility offering child health services (km)	5.8	1.1^§^
Facility quality index		
Lowest	8.4	22.3
Low	7.8	26.4
Middle	5.6	12.8
High	20.8	21.4
Highest	57.4	17.1
Percent public facility	83.5	22.4
Percent public hospital	48.3	0.9
Percent private facility	16.5	77.6

Mean values and percentages are weighted. Statistics not computed in instances where percent facility visited or nearest facility totals 100. Visited facility significantly differs from bypassed nearest facility

*p < 0.10; **p < 0.05; ^§^ *p* < 0.01

Facility quality and bypassing were also examined by poverty status. Among the 5217 women surveyed at endline, 45.5% were classified as poor. Among the 800 women who delivered at an MLE facility in the past 2 years, a higher percentage of non-poor women bypassed the nearest facility for delivery compared to poor women (90.3 vs 85.5%; *p* = 0.081). Among women bypassing the nearest facility offering delivery, poor women traveled an average of 4.5 km further than non-poor women (Table 4). Non-poor women bypassed to higher quality facilities and both poor and non-poor women bypassed to public hospitals. The majority of poor and non-poor women lived closest to a private facility that they bypassed, but more than twice as many non-poor women attended a private facility (33.4%) compared to poor women (12.9%).

**Table 4:** Quality comparison of MLE facility visited and MLE facility bypassed for each service received stratified by poor and non-poor

Women who reported a facility delivery in the past 2 years at endline	Poor bypass *N* = 325		Non-poor bypass *N* = 377	
	Facility visited	Nearest facility	Facility visited	Nearest facility
Mean distance to facility offering delivery (km)	12.4	2.0^§^	7.9	1.3^§^
Facility quality index				
Lowest	0.3	5.2	0.0	4.8^§^
Low	0.3	18.6	0.0	15.3
Middle	3.7	6.4	8.3	6.4
High	12.8	29.8	9.0	31.5
Highest	83.1	40.0	82.8	42.0
Percent public facility	87.1	36.1	66.6	37.1
Percent public hospital	76.8	1.7^§^	63.1	9.6^§^
Percent private facility	12.9	63.9	33.4	62.9
Women using facility-based contraception at endline	Poor bypass *N* = 663		Non-poor bypass *N* = 564	
	Facility visited	Nearest facility	Facility visited	Nearest facility
Mean distance to facility offering facility-based methods (km)	5.5 km	1.2 km^§^	6.9 km	0.9 km^§^
Facility quality index, %				
Lowest	3.3	28.7^§^	3.7	25.4**
Low	7.8	21.5	7.4	19.3
Middle	17.0	11.5	17.2	13.6
High	22.6	21.7	16.8	22.1
Highest	49.3	16.5	54.8	19.7
Percent public facility	82.0	18.6^§^	74.0	23.4
Percent public hospital	46.5	1.2**	47.0	2.1^§^
Percent private facility	18.1	81.4^§^	26.0	76.6
Percent private offer some free methods	3.2	10.0	3.1	7.0**
Mean total facility-based methods offered	3.4	2.6^§^	3.5	2.6^§^
Percent facility-based method stockout past year	29.0	17.5	31.2	25.3
Women who reported a child health visit in the past 3 months at endline	Poor bypass *N* = 497		Non-poor bypass *N* = 443	
	Facility visited	Nearest facility	Facility visited	Nearest facility
Mean distance to facility offering child health services (km)	5.4 km	1.2 km^§^	6.3 km	1.0 km^§^
Facility quality index				
Lowest	7.8	22.8	8.9	21.8
Low	7.0	26.6	8.5	26.2
Middle	6.7	11.6	4.6	14.0
High	26.1	23.5	15.7	19.5
Highest	52.4	15.6	62.3	18.5
Percent public facility	88.9	19.0	78.3	25.6
Percent public hospital	48.7	0.6*	47.9	1.2
Percent private facility	11.2	81.0	21.7	74.4

Mean values and percentages are weighted. Visited facility significantly differs from bypassed nearest facility

*p < 0.10; **p < 0.05; ^§^ *p* < 0.01

Among the 1467 women using facility-based contraception at endline, a higher percentage of non-poor women bypassed the nearest facility compared to poor women (86.5 vs 81.7%; *p* = 0.031). Both poor and non-poor women bypassed to higher quality facilities and nearly half visited public hospitals (Table 4). A higher percentage of poor women visited other public health centers (35.5%) compared to non-poor women (27%), while a higher percentage of non-poor women (26.0%) visited private facilities compared to poor women (18.1%). Non-poor women traveled slightly further than poor women. Both poor and non-poor women visited facilities that offered significantly more facility-based contraceptive methods.

Of the 940 women seeking child health services, a similar percentage of non-poor and poor women bypassed the nearest facility (84.9 vs 83.6%; *p* = 0.622). Poor and non-poor women traveled similar distances to visit a facility (Table 4). Similar percentages of poor and non-poor women chose to bypass to public hospitals, with a higher percentage of non-poor women (21.7 vs 11.2%) choosing to bypass to a private facility.

Logistic regression results examining which women bypass suggest that women living further from the nearest facility offering delivery have increased odds of bypassing (Table 5: model 1). Surprisingly, the quality of the nearest facility was not associated with bypassing for delivery. Living near a hospital, and whether the facility was public, significantly reduced the odds that a woman would bypass for delivery. Poor women were significantly (*p* = 0.01) less likely than non-poor women to bypass the nearest facility (AOR 0.35; 95%CI 0.17, 0.73).

**Table 5:** Logistic regression results measuring association between bypassing behavior and facility quality and distance

Models and variables of interest	Odds ratio	95%CI	*p* value
Model 1: Facility bypassing for delivery in past 2 years (*N* = 784)			
Variables for nearest facility offering delivery services			
Distance (km)	1.42	1.04, 1.93	0.03
Quality index			
Highest	Ref	Ref	Ref
High	1.24	0.55, 2.80	0.60
Middle	0.84	0.22, 3.30	0.81
Low	4.02	0.61, 26.5	0.15
Lowest	0.73	0.09, 5.95	0.77
Facility level			
Dispensary	Ref	Ref	Ref
Health center	0.08	0.01, 1.13	0.06
Hospital	0.01	0.001, 0.19	< 0.01
Public	0.16	0.09, 0.30	< 0.01
Model 2: Facility bypassing for current use of facility-based contraceptives (*N* = 1046)			
Variables for nearest facility offering facility-based methods			
Distance (km)	1.50	1.22, 1.85	< 0.01
Quality index			
Highest	Ref	Ref	Ref
High	0.48	0.20, 1.15	0.10
Middle	0.34	0.14, 0.83	0.02
Low	0.80	0.33, 1.97	0.63
Lowest	2.44	0.82, 7.21	0.11
Facility level			
Dispensary	Ref	Ref	Ref
Health center	1.21	0.65, 2.26	0.55
Hospital	0.56	0.22, 1.47	0.24
Public	0.07	0.04, 0.12	< 0.01
Stockout of facility-based contraceptive methods in past year	1.80	0.94, 3.45	0.08
Total facility-based contraceptive method types offered	0.51	0.22, 1.15	0.12
Private facility offers some methods free	0.65	0.22, 1.88	0.43
Model 3: Facility bypassing for child health visit in the past 3 months (*N* = 1078)			
Variables for nearest facility offering child health services			
Distance (km)	1.48	1.14, 1.91	< 0.01
Quality index			
Highest	Ref	Ref	Ref
High	0.68	0.31, 1.46	0.32
Middle	0.55	0.24, 1.26	0.16
Low	1.25	0.57, 2.76	0.58
Lowest	1.54	0.57, 4.17	0.40
Facility level			
Dispensary	Ref	Ref	Ref
Health center	0.36	0.18, 0.72	< 0.01
Hospital	0.06	0.02, 0.17	< 0.01
Public	0.04	0.02, 0.07	< 0.01

All models adjusted for individual level demographics including age, marital status, wealth, education, and city of residence

Living near a facility with a quality index rank of middle was negatively associated with bypassing for facility-based contraceptives (Table 5: model 2). Living further from the nearest facility was also associated with bypassing. Women who lived near a public facility were less likely to bypass. The level of facility was not associated with bypassing for facility-based contractive methods. Women who were poor were less likely to bypass for facility-based methods (AOR 0.53; 95%CI 0.31, 0.90; *p* = 0.02).

Living near a public facility significantly reduced the odds of bypassing the nearest facility (Table 5: model 3). Facility level was also negatively associated with bypassing as the odds of bypassing the nearest facility decreased if it was a health center or hospital compared to a dispensary. Similar to the other models, increased distance to the nearest facility was positively associated with bypassing. Being poor was not associated with bypassing for child health services.

## Discussion

The majority of women bypassed the nearest facility for delivery, facility-based contraception, and child health visits. Heterogeneity of facility availability exists by service sought. On average, as expected, women who bypassed for delivery traveled further and had the fewest available facility options within walking distance of their residential cluster compared to women bypassing for facility-based contraception or child health visits. This reflects that not all facilities offer delivery services and many women in our survey delivered in public hospitals which are fewer in number. When stratified by poverty status, poor women were less likely to bypass for delivery and those who did traveled 4.5 km further than non-poor women. Poor women were less likely to bypass for facility-based methods compared to non-poor women; however, poor and non-poor women seeking facility-based contraception or child health services traveled similar distances.

Among women who bypassed the nearest available facility for delivery, two thirds visited a public hospital. Logistic regression results suggest that the quality of the nearest facility does not influence the decision to bypass because public hospitals are preferred for delivery to other high quality health centers or private hospitals. The high percentage of women bypassing to public hospitals for delivery does not align with previous research that found women perceive public hospitals as unfriendly to poor patients, and unaffordable with added service and equipment fees [29]. However, Keesara et al. [35] noted that women view public hospitals as better equipped which could help explain the high use. Further, cost may be less of an issue because the survey took place following Kenya’s policy changes removing patient fees for delivery services [2].

Approximately 47% of women bypassing for facility-based contraception visited a public facility. The logistic regression results suggest that women are less likely to bypass for facility-based contraceptives if the facility closest to their home is of middle quality compared to lowest quality and is public. The level of the nearest facility was not associated with bypassing. It is possible that women choose to visit the closest facility if it is public, regardless of facility type, because facility-based methods are free in the public sector.

Among women who bypassed for child health services, 48% visited a public hospital. Women who lived near a health center or hospital, and public facility were less likely to bypass for child health services. The quality of the nearest facility was not associated with bypassing for child health services. The results suggest that women prefer higher level public facilities when they are seeking routine care such as immunizations, or acute care such as malaria treatment. Women may feel more comfortable taking their child to a higher level facility and prefer public facilities because they offer child health services for free.

The majority of women lived closest to a private facility regardless of service sought. Women bypassed smaller, private facilities and traveled further for public hospitals, likely due to cost. However, 18 to 24% of women chose private facilities for each service, with the highest percentage among women seeking delivery. Heterogeneity was observed by poverty status with non-poor women visiting private facilities nearly three times as often for delivery and twice as often for child health visits compared to poor women. Private facility use by some poor women, however, was not surprising despite cost, as studies from Nairobi slums found that women seeking care for sick children will visit private facilities within the slum as this is often the only option [22]. Women have also cited preferences for obtaining family planning services from private facilities to avoid public facility wait times [35], and delivering at private facilities because they are located within the slum eliminating distance and transport costs and logistics [24].

The high percentage of visited facilities with stockouts was unexpected. Women seeking facility-based contraception bypassed to facilities with significantly more method options as well as more reported stockouts in the past year. Results were similar among poor and non-poor women seeking facility-based methods. It is possible that the stockouts were due to high demand from women seeking higher quality facilities.

A strength of this study is our unique, representative sample of five urban sites that captures information on several maternal and child health needs allowing us to examine heterogeneity of facility access and selection across delivery, family planning, and child health services. Few studies have taken this approach [15]. It is important to understand what factors influence facility selection which can be informative for improving repeated facility use on the continuum of care (e.g., antenatal care, delivery, postnatal care, family planning, and child growth monitoring). Encouraging consistent facility use can benefit health outcomes and is often preferred when seeking child health services [17] and could encourage delivery at facilities where antenatal visits occur [29].

Another strength of our study is the implementation of a facility survey that captured service quality information and allowed us to directly link women to facilities where they sought care, which is not often manageable with large population-based studies [30]. A limitation of our facility survey is that a census was undertaken in a subset of participating cities, because there were too many facilities in Mombasa and Nairobi to identify and include in the survey. However, the facility survey does include a census of public facilities in all cities, as well as private facilities that were frequently reported by women at baseline as facilities where they obtained family planning and maternal and child health services. In addition, sensitivity analyses suggest that the partial census in our two larger cities did not influence results.

Using precise GPS location information allowed us to accurately measure and compare distance between visited and nearest available facilities offering each service. While we were unable to estimate travel time due to limited available road network data in our study areas, we were able to measure straight-line distance which identified differences in distance between visited and nearest facilities, and can more accurately represent distance traveled than self-report [36]. We were also unable to capture transportation mode (i.e., walk, taxi) for this study. Therefore, our analysis assumes that transportation mode does not influence facility selection. This is a limitation of our study as transportation mode has been found to influence facility delivery [18] and non-poor women may have greater access to main roads to hire cars or taxis compared to poor women, reducing their travel burden [26]. It is possible that participant responses to facilities visited are subject to recall bias as child health visits occurred in the past 3 months and delivery was within 2 years. However, by linking the facility survey data, we were able to ensure that the facilities women reported visiting did in fact offer those services. Another limitation of our study is the lack of available cost data. We were able to account for free facility-based contraceptives offered at some private facilities, and all public facilities offer services for free, but we were unable to capture the effect of cost for delivery or child health services at private facilities.

An examination of facility choice across the three service delivery options provides an interesting perspective of women’s (and families’) choices about health care in urban Kenya. Many studies have focused on the decision to seek care but few have examined facility selection [8, 15, 17, 18, 20, 22]. Across all three models, we find a preference for public sector facilities. As discussed above, this likely relates to free services in these settings [2]. Across all of the models, we found that when the distance to the closest facility is greater, the women are more likely to bypass. This likely reflects that once a woman has to travel to a facility that is not close to her home, she is acting on her preference for a higher quality (or public) facility. When the nearest facility is closer to her home, she may weigh her options differently before bypassing.

Our study findings suggest that women in five cities in Kenya prefer public hospitals to other facility types and are willing to travel further to obtain services at public hospitals. This may be related to free services available at public sector facilities for delivery, facility-based contraception, and child health services. Over time, it will be important to examine service quality and availability in public sector facilities with reduced or eliminated user fees, and whether it lends itself to a continuum of care where women prefer the same facility for multiple services. There is a possibility that this increases use, but it could also lead to over-burdened facilities and result in poorer quality services. As programs seek to reach women (and families) with family planning and maternal and child health services, they need to ensure that services offered are of a high enough quality to attract clients and meet the clients’ varying considerations of quality, distance, cost, and services offered. Future studies need to continue to examine where women go for delivery, facility-based contraception, and child health services to capture temporal trends and make recommendations for future policies and programs to improve women’s and families’ health and well-being.

## Electronic Supplementary Material

ESM 1 (DOCX 28.1 kb)

## Footnotes

1 Facilities missing patient estimates were assigned the average number of patients by facility type.
