# Supplementary material for: The Role of Distance and Quality on Facility Selection for Maternal and Child Health Services in Urban Kenya
Source: J Urban Health. 2017 Dec 21;95(1):1–12. doi: 10.1007/s11524-017-0212-8 (PMC5862698; doi:10.1007/s11524-017-0212-8)
Supplement: Supplementary file 1 — (DOCX 28.1 kb) [file 11524_2017_212_MOESM1_ESM.docx]

**Supp A: Quality characteristics offering delivery, facility-based methods, or child health services stratified by facility type**

| **Facility characteristics** | **Public hospital**  **N=16** | **Private hospital**  **N=29** | **Public health center**  **N=48** | **Private health center**  **N=190** | **Dispensary***  **N=46** |
| --- | --- | --- | --- | --- | --- |
| **Service offered** |  |  |  |  |  |
| Delivery | 93.8 | 86.2 | 45.8 | 38.5 | 34.8 |
| Long acting methods | 100 | 79.3 | 100 | 96.0 | 100 |
| Child health services | 100 | 96.6 | 100 | 94.7 | 100 |
| **Accessibility** |  |  |  |  |  |
| Open 7 days per week | 93.8 | 93.1 | 52.1 | 65.8 | 13.0 |
| Open 24 hours | 93.8 | 89.7 | 41.7 | 42.0 | 4.5 |
| **Staff** |  |  |  |  |  |
| Mean total physicians per 1,000 | 0.5 | 2.9 | 0.0 | 0.4 | 0.2 |
| Mean total nurses per 1,000 | 48.3 | 44.6 | 8.9 | 15.8 | 6.2 |
| Mean total community health  workers per 1,000 | 0.9 | 0.4 | 1.0 | 1.2 | 1.6 |
| **Infrastructure** |  |  |  |  |  |
| Electricity | 100 | 100 | 95.8 | 98.2 | 84.8 |
| Piped water | 100 | 100 | 87.5 | 78.8 | 65.2 |
| Functional private exam room | 100 | 96.6 | 95.8 | 90.3 | 80.4 |
| Functional exam table/couch | 100 | 96.6 | 91.7 | 81.3 | 95.7 |
| **Hygiene resources** |  |  |  |  |  |
| Liquid soap | 93.8 | 89.7 | 93.8 | 95.6 | 87.0 |
| Antiseptic | 100 | 96.3 | 87.5 | 91.2 | 78.3 |
| Latex gloves | 100 | 100 | 97.9 | 96.5 | 65.7 |
| Functional sterilizer/autoclave | 100 | 96.4 | 85.4 | 74.2 | 63.0 |
| **Mean quality index score** | 4.7 | 4.5 | 3.0 | 2.9 | 2.0 |

Note: All values represent percent of facilities reporting a characteristic unless otherwise specified

*Includes 45 public dispensaries and one private dispensary

**Supplement B: Sensitivity analysis restricted to Kisumu, Kakamega, and Machakos where a census of all facilities was conducted**

| Facility characteristics | Facility visited | Nearest facility | |
| --- | --- | --- | --- |
| **Delivery reported in last 2 years (N=437)** | | | |
| Mean distance to facility offering delivery (km) | 10.8 | 2.0^§^ | |
| Facility quality index |  |  | |
| Lowest | 0.0 | 4.1 | |
| Low | 0.2 | 20.1 |  |
| Middle | 1.7 | 3.7 |  |
| High | 3.6 | 18.5 |  |
| Highest | 94.6 | 53.3 |  |
| Percent public facility | 81.3 | 44.5 |  |
| Percent public hospital | 78.1 | 3.5^§^ |  |
| Percent private facility | 18.7 | 55.5 |  |
| **Facility-based contraceptive use at endline (N=377)** | | |  |
| Mean distance to facility offering long-acting methods (km) | 7.1 | 1.2^§^ |  |
| Facility quality index, *%* |  |  |  |
| Lowest | 1.0 | 30.1^§^ |  |
| Low | 5.0 | 21.3 |  |
| Middle | 16.3 | 11.6 |  |
| High | 17.1 | 17.7 |  |
| Highest | 60.6 | 19.3 |  |
| Percent public facility | 80.3 | 19.7* |  |
| Percent public hospital | 56.4 | 1.0^§^ |  |
| Percent private facility | 19.7 | 80.3* |  |
| Percent private facility offering some long-acting methods free | 2.9 | 8.2** |  |
| Mean total long-acting methods offered | 3.5 | 2.5^§^ |  |
| Percent report long-acting method stockout in past year | 33.8 | 17.7** |  |
| **Reported child health visit in past 3 months (N=679)** | | |  |
| Mean distance to facility offering child health services (km) | 6.4 | 1.2^§^ |  |
| Facility quality index |  |  |  |
| Lowest | 4.8 | 26.4 |  |
| Low | 6.9 | 27.0 |  |
| Middle | 10.0 | 12.7 |  |
| High | 78.3 | 16.9 |  |
| Highest | 0.0 | 17.0 |  |
| Percent public facility | 86.1 | 22.9 |  |
| Percent public hospital | 56.6 | 0.5** |  |
| Percent private facility | 13.9 | 77.1 |  |
